# Adult Medulloblastoma: Occurrence of a Rare Event

**DOI:** 10.7759/cureus.3000

**Published:** 2018-07-18

**Authors:** Mariya Kristeva, Andrey Suprun, Ejaz Ghaffar, Carolina Wallis

**Affiliations:** 1 University of Central Florida College of Medicine, Orlando, USA; 2 Medical Student, University of Central Florida College of Medicine, Orlando, USA; 3 Department of Internal Medicine, Osceola Regional Medical Center, Orlando, USA; 4 Department of Pathology, Osceola Regional Medical Center, Orlando, USA

**Keywords:** medulloblastoma, adult

## Abstract

Medulloblastoma is the most common type of aggressive pediatric primary brain malignancy. This case describes a 45-year-old Hispanic male with no significant past medical history who presented to the emergency department (ED) complaining of 15 days of 10/10 intractable headaches with one day of lightheadedness, confusion, and loss of balance. An urgent magnetic resonance imaging (MRI) of the brain revealed a 4.1 x 3.3 x 3.2 cm mass at the cerebellum, exerting a mass effect on the brainstem and mild tonsillar herniation. A pre-surgical physical exam revealed only a positive Babinski sign bilaterally with normal proprioception and cerebellar function. The intraoperative report concluded an undifferentiated neoplasm with a histological differential diagnosis of medulloblastoma, ependymoma, or other neuroepithelial neoplasms, suggesting a referral to a tertiary care center for further evaluation of the mass. Postsurgical complications included a severe vasogenic edema, causing obstructive hydrocephalus treated with frontal ventricular drainage, signs of meningitis treated with antibiotics, and hyponatremia. This case describes a rare occurrence of medulloblastoma in an adult patient, which required prompt diagnosis and urgent life-saving treatment.

## Introduction

Medulloblastoma is the most common, aggressive, invasive, malignant embryonal tumor of the cerebellum with a preferential manifestation in children. In the United States, 2200 children are diagnosed with a brain tumor of which each medulloblastoma accounts for 22% of all childhood primary tumors [1]. However, in adults, medulloblastoma account for less than 1% of adult intracranial tumors, with an incidence rate of about 0.5 per million [2-3]. The reason for the high incidence of medulloblastoma in children (one to nine years old) as compared to adults is the embryonal origin of the tumor, which is rare after the fifth decade of life [4]. Previous reports have identified differences in the cell of origin, tumor cell differentiation, and pathological features between childhood and adult medulloblastoma [5]. Specifically, Sonic hedgehog (SHH) pathway activation is a common feature of adult medulloblastoma and Al-Halabi et al. found SHH-driven tumorigenesis in more than 80% of cases of adult medulloblastoma [6]. Additionally, CDK6 amplification, 10q loss, and 17q gain mutations are the most useful prognostic markers in adult medulloblastoma. In contrast, MYC amplification is the most important prognostic marker in pediatric medulloblastoma and is rare in adult variants [7]. This case is unique because adult medulloblastoma occurrence is very rare and the diagnosis requires histopathological and immunohistochemical analyses, which is available only at tertiary centers.

## Case presentation

**Figure 1:**
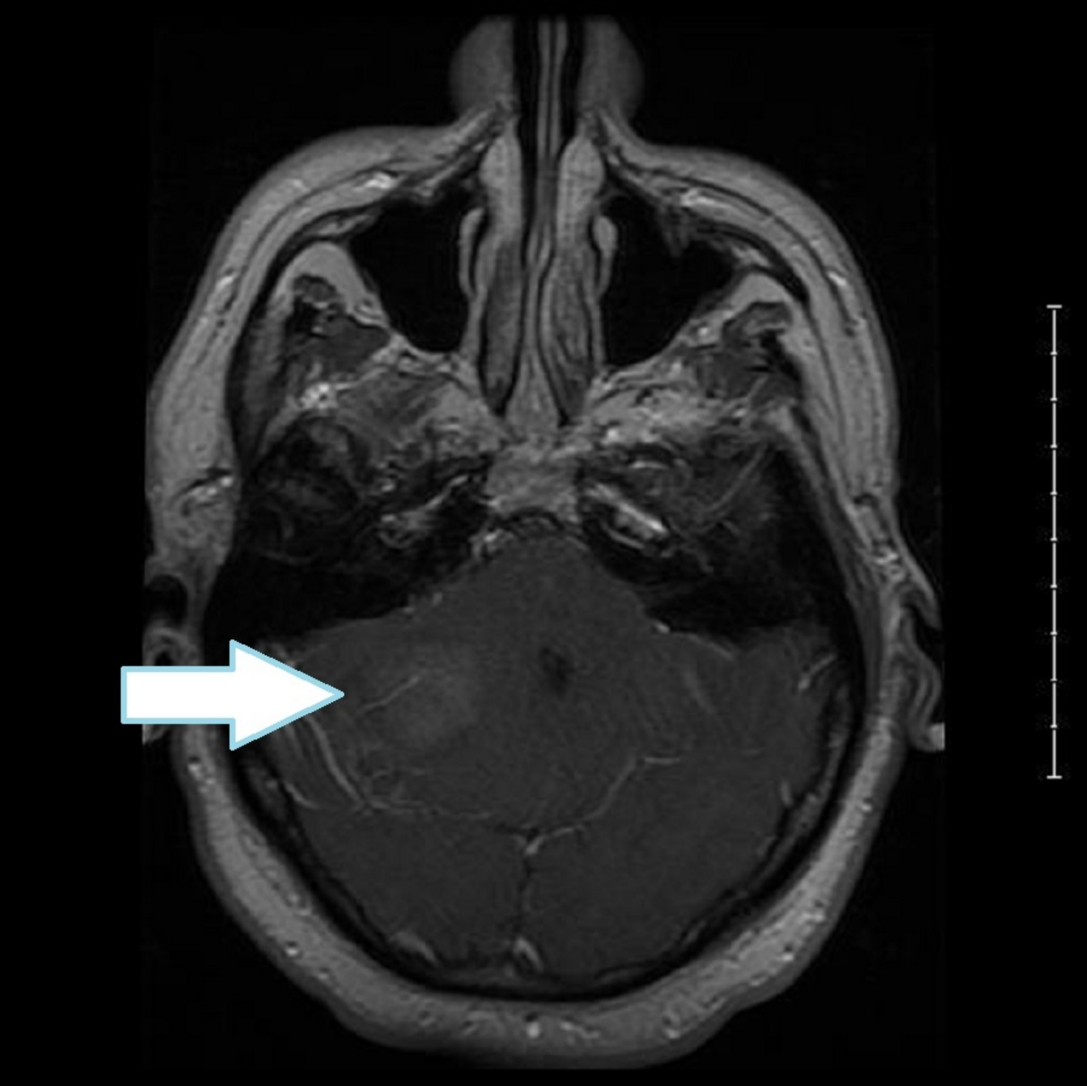
Preoperative MRI of brain with contrast post-axial T1 showing a hyper-dense posterior fossa tumor on the right side with mass effect and obstructive hydrocephalus MRI: magnetic resonance imaging

A 45-year-old male without a significant past medical history presented to the emergency department (ED) as a walk-in, complaining of 10/10 intractable headaches with lightheadedness, confusion, and loss of balance, starting one day before presentation to the ED. A computed tomography (CT) scan of the head done in the ED revealed a hyperdense lesion on the right at the level of the peri- pontine cistern and magnetic resonance (MRI) of the brain done for a further evaluation of the lesion revealed a 4.1 x3.3x 3.2 cm mass with mild tonsillar herniation and a mass effect on the brainstem (Figure 1 ).

**Figure 2:**
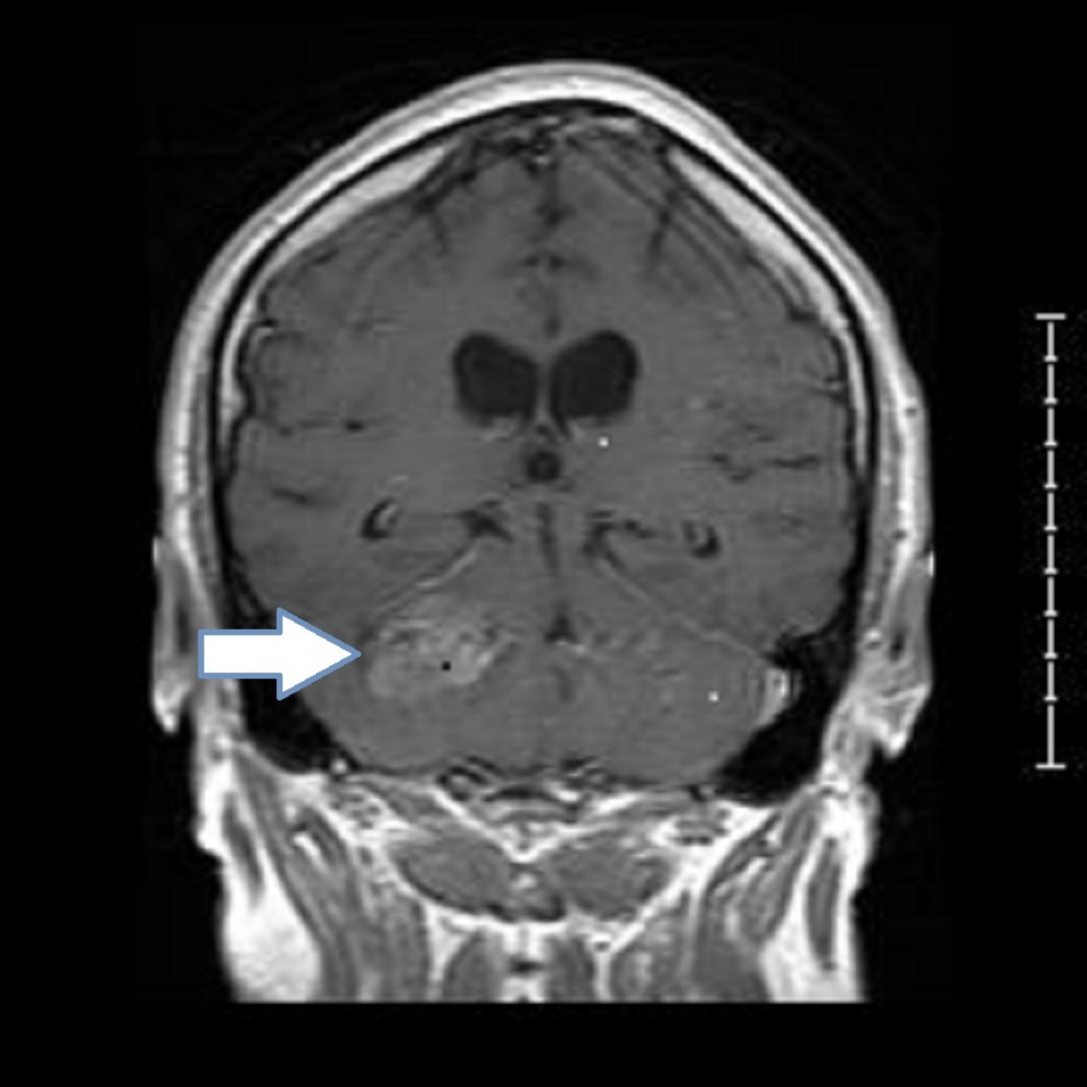
Preoperative MRI of brain with contrast post-COR T1 showing a posterior fossa tumor on the right with asymmetry of the cerebellar soft tissues with increased density

MR signal post-COR T1 revealed a hyperintense cortical grey matter lesion with a patchy heterogenous enhancement due to possible hemorrhage or necrosis (Figure 2).

On examination, he continued to complain of headaches, with the only pertinent physical findings being a positive Babinski sign bilaterally. The physical exam was negative for loss of proprioception and loss of cerebellar function (finger-to-nose test). Consequently, he was admitted for further work-up. Since an adult brain neoplasm is more likely to be a metastasis rather than a primary malignancy, CT scans of the chest, abdomen, and pelvis were obtained. The scans identified a right thyroid nodule but no lung mass on CT of the chest, and the abdomen was free of masses except for a 2.3 cm renal cyst. He had a right suboccipital craniotomy on Day 2 of admission. The intraoperative report concluded an undifferentiated neoplasm with a histological differential diagnosis of medulloblastoma, ependymoma, or other neuroepithelial neoplasms. Additionally, the CD45 marker was positive, which raised the differential diagnosis of a lymphoproliferative disorder. The case was referred for consultation to a tertiary care center, which made the diagnosis of classic medulloblastoma, world health organization (WHO) grade IV. The tumor was composed of densely packed cells with round to oval highly hyperchromatic nuclei surrounded by scanty cytoplasm most consistent with classic medulloblastoma with non-desmoplastic nodules. He subsequently developed vasogenic edema that completely occluded his fourth ventricle, producing obstructive hydrocephalus and required the emergent insertion of a frontal ventricular drain into the right lateral ventricle. He later developed a fever, headache, and neck stiffness suggestive of meningitis. Lumbar puncture was deferred due to raised intracranial pressure, and the patient was started on empiric antibiotics. Leukocytosis resolved and fever subsided. Additionally, he developed hyponatremia with urine osmolality of 384, urine sodium of 24, and serum osmolality of 284 with a differential diagnosis of syndrome of inappropriate anti-diuretic hormone (SIADH), cerebral salt wasting, or reactive elevation of the anti-diuretic hormone secondary to surgery. He was started on sodium chloride tablets, demeclocycline, and fluid restriction. Post-surgery imaging showed no metastasis and radiation oncology recommended follow-up in a tertiary center.

## Discussion

In adults above the age of 40, metastatic brain tumors account for more than half of all brain tumors. A statistical report of primary brain tumors states that medulloblastoma and other embryonal tumors are only common in age group zero-four years old with an incidence of 0.32 per 100,000 population and drop to 0.02 per 100,000 population in children age five–nine years. To date, there are only a few case reports published in the literature on medulloblastoma in the older age group [5].

Most patients present with headaches and ataxia or other nonspecific symptoms of increased intracranial pressure [6]. Additionally, an adult medulloblastoma has a variable and nonspecific presentation on imaging investigation [8]. Furthermore, tumor biology and the prognostic impact of adult medulloblastoma differ considerably from pediatric medulloblastoma [9]. The low incidence of medulloblastomas in adults and the uncertain correlation of adult and pediatric medulloblastoma prognostic and biological factors should stimulate research that can help shape the future standardization of procedures to treat this rare-to-diagnose tumor in adults.

Our case illustrated the potential for developing an embryonal tumor late in adulthood. Adults with medulloblastoma have a worse prognosis compared with children and early diagnosis is paramount since disseminated or metastatic disease at the time of diagnosis is associated with a particularly poor prognosis [10]. The delayed complications of medulloblastoma can have a profound effect on quality of life and mortality and increased recognition is critical for the institution of appropriate and timely therapy.

## Conclusions

Adults with medulloblastoma have a worse prognosis compared with children and early diagnosis is paramount since disseminated or metastatic disease at the time of diagnosis is associated with a particularly poor prognosis. Though adult medulloblastomas are rare, there are distinct imaging biomarkers that are different from those occurring in children. Delayed complications of medulloblastoma can have a profound effect on quality of life and mortality and increased recognition is critical for the institution of appropriate and timely therapy.
